# Defining ICD-10 surrogate variables to estimate the modified frailty index: a Delphi-based approach

**DOI:** 10.1186/s12877-022-03063-x

**Published:** 2022-05-13

**Authors:** Ashwin Subramaniam, Ryo Ueno, Ravindranath Tiruvoipati, Jai Darvall, Velandai Srikanth, Michael Bailey, David Pilcher, Rinaldo Bellomo

**Affiliations:** 1 Department of Intensive Care, Peninsula Health, Frankston, Victoria Australia; 2 Peninsula Clinical School, Monash University, Frankston, Victoria Australia; 3 Australian and New Zealand Intensive Care Research Centre, Department of Epidemiology and Preventive Medicine, Monash University, Melbourne, Victoria Australia; 4 Department of Intensive Care, Eastern Health, Box Hill, Victoria Australia; 5 Department of Intensive Care, Royal Melbourne Hospital, Melbourne, Victoria Australia; 6 Department of Critical Care, The University of Melbourne, Melbourne, Victoria Australia; 7 Department of Geriatric Medicine, Peninsula Health, Frankston, Victoria Australia; 8 National Centre for Healthy Ageing, Melbourne, Australia; 9 Department of Intensive Care, Alfred Hospital, Melbourne, Victoria Australia; 10 Centre for Outcome and Resource Evaluation, Australian and New Zealand Intensive Care Society, Melbourne, Victoria Australia; 11 Department of Intensive Care, Austin Hospital, Heidelberg, Victoria Australia

**Keywords:** definition, modified frailty index, mFI, International Statistical Classification of Diseases and Related Health Problems Tenth Revision, ICD-10 codes

## Abstract

**Background:**

There are currently no validated globally and freely available tools to estimate the modified frailty index (mFI). The widely available and non-proprietary International Statistical Classification of Diseases and Related Health Problems, Tenth Revision (ICD-10) coding could be used as a surrogate for the mFI. We aimed to establish an appropriate set of the ICD-10 codes for comorbidities to be used to estimate the eleven-variable mFI.

**Methods:**

A three-stage, web-based, Delphi consensus-building process among a panel of intensivists and geriatricians using iterative rounds of an online survey, was conducted between March and July 2021. The consensus was set a priori at 75% overall agreement. Additionally, we assessed if survey responses differed between intensivists and geriatricians. Finally, we ascertained the level of agreement.

**Results:**

A total of 21 clinicians participated in all 3 Delphi surveys. Most (86%, 18/21) had more than 5-years’ experience as specialists. The agreement proportionately increased with every Delphi survey. After the third survey, the panel had reached 75% consensus in 87.5% (112/128) of ICD-10 codes. The initially included 128 ICD-10 variables were narrowed down to 54 at the end of the 3 surveys. The inter-rater agreements between intensivists and geriatricians were moderate for surveys 1 and 3 (κ = 0.728, κ = 0.780) respectively, and strong for survey 2 (κ = 0.811).

**Conclusions:**

This quantitative Delphi survey of a panel of experienced intensivists and geriatricians achieved consensus for appropriate ICD-10 codes to estimate the mFI. Future studies should focus on validating the mFI estimated from these ICD-10 codes.

**Trial registration:**

Not applicable.

**Supplementary Information:**

The online version contains supplementary material available at 10.1186/s12877-022-03063-x.

## Keypoints

- There was an 87.5% consensus in the quantitative Delphi survey to define the ICD-10 variables required to estimate the modified frailty index (mFI) from the 21-panel members that comprised of geriatricians and intensivists, with moderate to a strong inter-rater agreement between the geriatricians and intensivists in all three surveys.
- 54 ICD-10 codes were considered necessary to estimate the 11 mFI variables.
- These ICD-10 codes can be further investigated for their validity in estimating frailty in the geriatric or intensive care setting.

Why does this paper matter?

In this study, we used a quantitative Delphi consensus process from a panel of twenty-one members comprising experienced geriatricians and intensivists, to define the 54 ICD-10 variables required as a surrogate to estimate the modified frailty index (mFI). We also demonstrated moderate to strong inter-rater agreement between geriatricians and intensivists in all three surveys. These ICD-10 codes could be further investigated for their validity in estimating frailty in the geriatric or intensive care setting. As ICD-10 coding is globally available and non-proprietary, the potential impact of this research on clinical care or health policy means that these 54 ICD-10 could be used to estimate an mFI equivalent to provide global frailty data in large populations at a national or multinational level.

## Introduction

Frailty is a clinically recognisable state of increased vulnerability due to aging-associated decline in reserve and function across multiple physiologic systems. Such people with frailty have a reduced ability to cope with acute stressors [1]. Patients with frailty are commonly admitted to intensive care units (ICU), and patients with frailty generally have poorer outcomes [2, 3]. As a result, frailty assessment is often recommended upon ICU admission [4], with a variety of frailty screening tools. Regrettably, frailty assessments are difficult to perform by critical care teams on a routine basis [5]. Administrative data have therefore been used as surrogates to assess the epidemiology of frailty retrospectively [6]. The modified frailty index (mFI) is one such administrative data-based surrogate.

The mFI was originally designed using data from the National Surgical Quality Improvement Program (NSQIP) database mapped to variables contained within the original 70-item Canadian Study of Health and Aging Frailty Index [7]. The score is based on eleven variables which encompass comorbidities, previous medical events, and functional capacity [8]. The mFI then categorizes patients as non-frail if score = 0; pre-frail if score = 1–2, or frail if the score is ≥3 [8–10], allowing for rapid derivation and automation. Although the mFI was originally created for patients having surgery [10, 11], it recently predicted an increased risk of hospital mortality, length of stay, and higher health resource utilization in a large Brazilian cohort of critically ill patients [8].

The mFI, however, has some limitations. In particular, it cannot be freely obtained outside of specific proprietary databases. Moreover, there are currently no validated globally and freely available tools to estimate the mFI. In theory, however, the widely available and non-proprietary International Statistical Classification of Diseases and Related Health Problems, Tenth Revision (ICD-10) coding could be used as a surrogate for the mFI. The frailty score identified, as a result, could be widely used on an international scale. However, the selection of which ICD-10 codes reflect the mFI items and can be used to estimate them remains challenging. Although several Delphi consensus processes have been performed to quantify frailty, no study to our knowledge, there were no studies with regards to the mFI. To address this issue, we undertook a Delphi consensus process to identify ICD-10 codes that geriatricians and intensivists would consider as representative of the eleven mFI items. Additionally, we assessed if survey responses differed between intensivists and geriatricians. Finally, we ascertained the level of agreement. We hypothesized that there would be a consensus amongst intensivists and geriatricians in determining the ICD-10 codes for the eleven mFI variables as an approximation.

## Materials and methods

### Ethics approval

This study was approved by The Research Governance of Peninsula Health Ethics Committee (HREC reference number AM/47502/PH-2021-251,553(v2)).

### Source of ICD-10 codes

Diagnosis Related Groups (DRG) is an admitted patient classification system that provides a clinically meaningful way of relating the number and type of patients treated in a hospital to the resources required by the hospital. Each DRG represents a class of patients with similar clinical conditions requiring similar hospital services. The ICD-10 codes were obtained from the Australian-Refined Diagnosis Related Groups (AR-DRG) upon patient discharge. Although a recent systematic review identified that hospital given that the 11-item mFI (listed in Supplementary Table 1), was derived from the original 70-item Canadian Study of Health and Aging Frailty Index, we used the NSQIP frailty index. The AR-DRG was screened for pertinent ICD-10 codes by a single author (AS). This was discussed and agreed upon with three other authors (RT, JD, and DP). The relevant ICD-10 codes that encompass the nine comorbidities of the mFI were included. However, the remaining two mFI variables (‘not independent of functional status’ and ‘impaired sensorium’) did not have readily available ICD-10 codes. Therefore, all possible ICD-10 codes for these variables were included for the Delphi consensus (Supplementary Table 2).

### The Delphi panel

The purpose of the Delphi panel was to reach a consensus on the ICD-10 variables required to calculate the mFI. The panel members comprised a combination of intensivists and geriatricians. The participation was voluntary and agreeing to participate was taken to indicate informed consent. No incentives were offered. All the panel members participated in this multi-step process. We collected basic demographic information on the type of specialist and their years of clinical experience as a specialist.

### The Delphi process

**Fig. 1:**
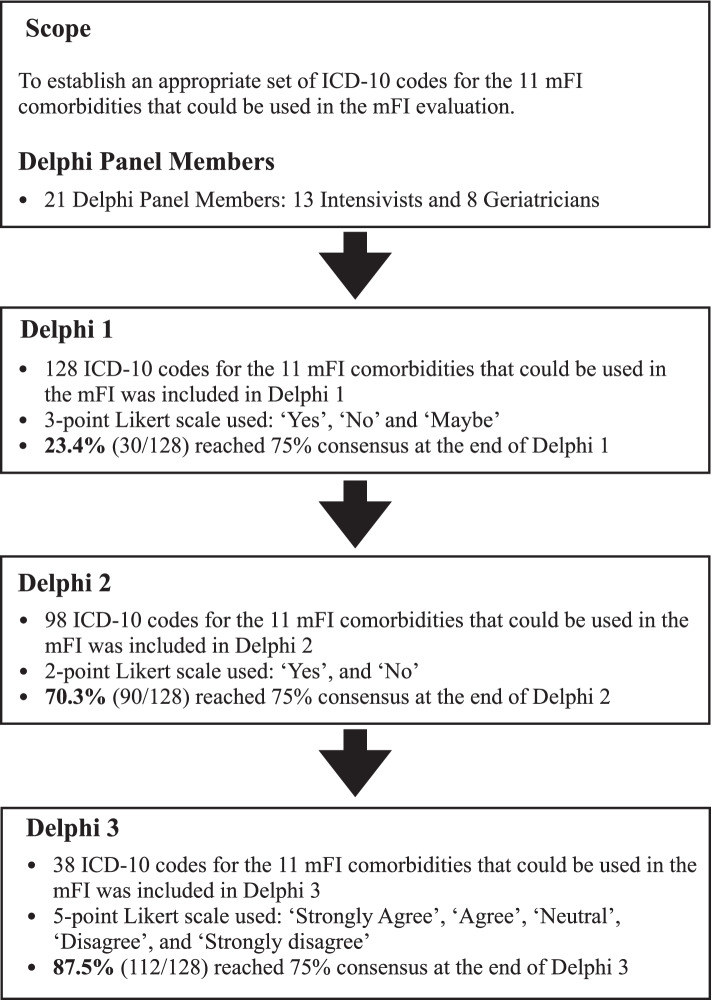
Flowchart to illustrate the stage of the Delphi process

Input from the panel was obtained using a 3-step Delphi Consensus-building process. Each step was comprised of a web-based survey. We also provided the weblink to the NSQIP database and definitions that were used for every variable [7], with results discussed in web-based meetings. The variables that reached consensus were removed in the subsequent Delphi survey. The details of the Delphi process are summarised in Fig. 1. The round 1 Delphi survey questionnaire comprised of 128 items, where the panel members were expected to mark the items on a 3-point Likert scale of ‘Yes’, ‘Maybe’, and ‘No’ (Supplementary Appendix 1). In Delphi round 2, the panel members were requested to mark the items on a two-point Likert scale, either as “Yes” or “No” (Supplementary Appendix 2). In round 3, the panel members were asked to rate the importance of the remaining ICD-10 variables using a 5-point Likert scale: ‘strongly disagree’, ‘disagree’, ‘neutral’, ‘agree’, and ‘strongly agree’ (Supplementary Appendix 3). We grouped the responses ‘strongly agree’, ‘agree’ and ‘neutral’ into one outcome and ‘disagree’ and ‘strongly disagree’ into another.

### Consensus

The consensus was set a priori at 75% overall agreement by the panel members for these ICD-10 codes. In cases where the panel did not reach at least 75% consensus to keep or remove an ICD-10 code, we included it in the subsequent survey. This level of agreement has been considered appropriate in previous Delphi studies [12]. This process was continued until at least 85% of the possible ICD-10 codes for each of the eleven variables reached a minimum of 75% consensus.

### Outcomes

The primary outcome was to establish an appropriate set of ICD-10 codes as a surrogate for the eleven items used in the mFI. The secondary outcome included group comparison to assess any differences between intensivists and geriatricians and to ascertain a level of agreement amongst them.

### Data analysis

Descriptive statistics were used to report participants’ demographic characteristics. Dichotomous and categorical data were described using frequencies and percentages. Fisher’s exact test was used for group comparisons between intensivists and geriatricians. We not only reported an overall combined comparison between intensivists and geriatricians but also comparisons for individual mFI variables. The measure of agreement between the two groups was analyzed using the Kappa statistic and was reported for each survey. We defined the Kappa (κ statistic) measure of an agreement value to be 0–0.20 as no agreement, 0.21–0.39 as minimal agreement, 0.40–0.59 as weak agreement, 0.60–0.79 as moderate agreement, 0.80–0.90 as strong agreement, and > 0.90 as almost perfect agreement [13]. All *p*-values reported were two-tailed and the threshold for statistical significance was set at *p* < 0.05. SPSS (version 27, IBM) was used for all analyses.

## Results

**Fig. 2:**
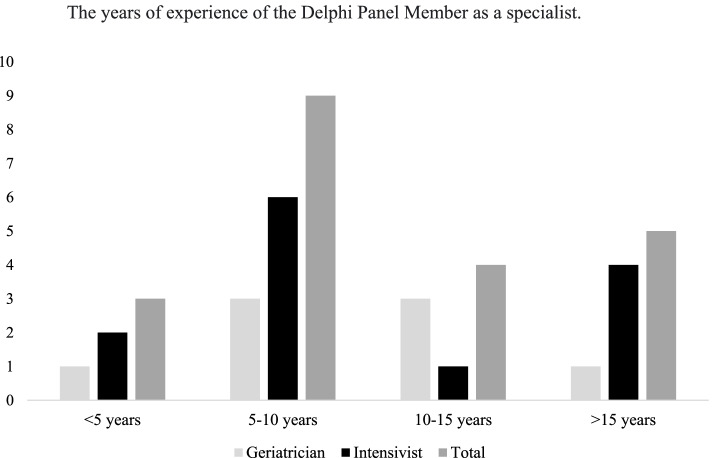
Number of Delphi Panel Members and their years of experience as a specialist

A total of 21 senior clinicians participated in all the 3 Delphi surveys as panel members. Most (86%, 18/21) had more than 5-years’ experience as a specialist, 43% had more than 10 years’ experience (Fig. 2).

### Primary outcome

**Fig. 3:**
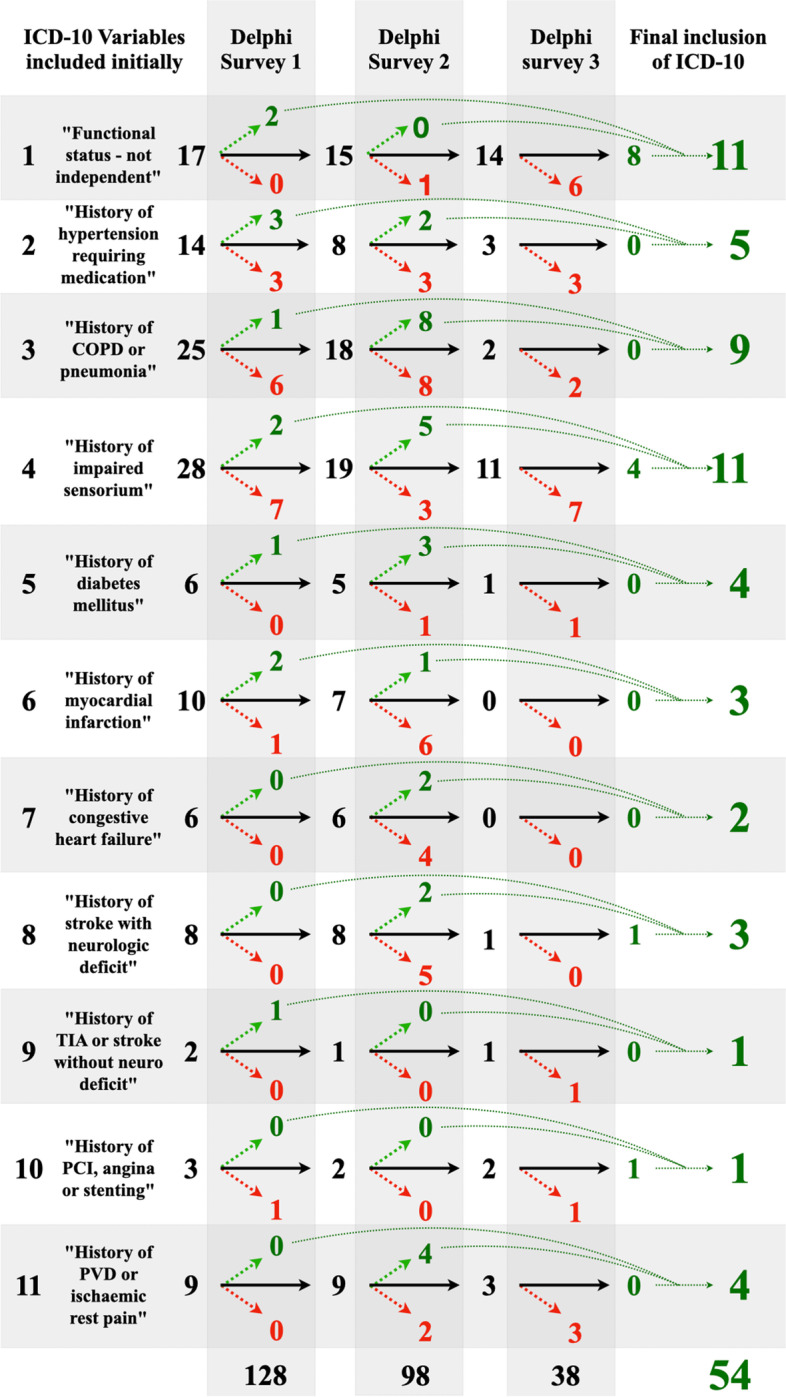
The three rounds of the Delphi consensus survey demonstrating how the 128 ICD-10 variables initially included, were narrowed down to 54 to determine the mFI. Numbers in red depict excluded variables after each Delphi round, while numbers in green depict included ICD-10 variables

Agreement proportionately increased with every Delphi survey. After the first survey, the panel had reached 75% consensus in 23.4% (30/128) of the ICD-10 codes. Following the second survey, a consensus was reached for 70.3% (90/128) of ICD-10 codes. This increased to 87.5% of codes (112/128) after the third survey. Figure 3 illustrates the three rounds of the Delphi consensus survey that demonstrated how the 128 ICD-10 variables initially included, were narrowed down to 54 (42.2%) to estimate the mFI (Table 1, Supplementary Tables 3, 4 and 5). The 16 ICD-10 variables (12.6%) that did not reach 75% consensus were also removed (Supplementary Table 6).

**Table 1:** ICD-10 variables included to determine modified frailty index following the three rounds of Delphi consensus survey

**Variable 1: “Functional status (not independent)”**	1. H54 – Blindness and low vision
	2. R26.0- R26.9 – Gait problems
	3. R27.0 -R27.9 – Lack of coordination
	4. R41 – Sarcopenia
	5. R41.81 – age-related cognitive decline
	6. R54 – age-related physical disability
	7. S72 – fracture of femur
	8. Z73 – Life management difficulty
	9. Z74.1 – Need for assistance
	10. Z73.6 – Activities of daily living dysfunction
	11. Z74 – Care-provider dependency
**Variable 2: “History of hypertension requiring medication”**	12. I10 – Essential hypertension
	13. I11 – Hypertensive heart disease
	14. I12 – Hypertensive kidney disease
	15. I13 – Hypertensive heart and kidney disease
	16. I15 – Secondary hypertension
**Variable 3: “History of chronic obstructive pulmonary disease or pneumonia”**	17. J12 – viral pneumonia
	18. J13 – pneumonia due to streptococcal pneumoniae
	19. J14 – pneumonia due to *Haemophilus influenzae*
	20. J15 – Bacterial pneumonia, not elsewhere classified
	21. J16 – Pneumonia due to other infectious organisms
	22. J17 – Pneumonia in diseases classified elsewhere
	23. J18 – Pneumonia, organism unspecified
	24. J43 – Emphysema
	25. J44 – Chronic obstructive pulmonary disease
**Variable 4: “History of impaired sensorium”**	26. A81.0 – Creuksfeld Jacob Disease
	27. F00-F03 – Delirium superimposed with dementia
	28. F01 – Vascular Dementia
	29. F04 – Organic Amnesia Syndrome
	30. F05 – Delirium
	31. F06 – Memory disturbance
	32. F10 – Amnesia related to Alcohol
	33. F11-F19 – Amnesia related to psychoactive agents
	34. G20 - Parkinson’s disease
	35. G30 – Alzheimer’s Dementia
	36. H35 – Macular degeneration
**Variable 5: “History of diabetes mellitus”**	37. E10 - Type 1 diabetes mellitus
	38. E11 - Type 2 diabetes mellitus
	39. E13 – Other specified diabetes mellitus
	40. E14 - Unspecified diabetes mellitus
**Variable 6: “History of myocardial infarction”**	41. I21 – Acute myocardial infarction
	42. I22 – Subsequent myocardial infarction
	43. I25 – Chronic ischaemic heart disease
**Variable 7: “History of congestive heart failure”**	44. I50 – Heart failure
	45. U80.2 – Chronic heart failure
**Variable 8: “History of stroke with neurologic deficit”**	46. I61 – Intracerebral haemorrhage
	47. I63 – Cerebral infarction
	48. I69 – Sequelae of cerebrovascular disease
**Variable 9: “History of TIA or stroke without neurological deficit”**	49. G45 – Transient cerebral ischaemic attacks and related syndromes
**Variable 10: “History of PCI, angina or stenting”**	50. I20 – Angina pectoris
**Variable 11: “History of Peripheral vascular disease or ischaemic rest pain”**	51. I70.2 - Atherosclerosis of arteries of extremities
	52. I73 - Peripheral vascular disease
	53. I77.9 - Peripheral arterial insufficiency
	54. I77.1 - Obliterative peripheral arteries

### Secondary outcome

Of the 21 Delphi panel members, 13 were intensivists and 8 were geriatricians. There was no difference in the levels of experience between the 2 groups (84.6% [11/13] vs. 87.5% [7/8]; *p* = 0.74). The overall inter-rater agreement between the intensivists and geriatricians was moderate for survey 1 (κ = 0.728); strong for survey 2 (κ = 0.811) and moderate agreement for survey 3 (κ = 0.780) (Table 2). When the individual variables were compared, the inter-rater agreement was consistently between moderate and almost perfect agreement (Table 2).

**Table 2:** Measure of Agreement between Intensivists and Geriatricians

		Delphi survey 1 κ statistic^	Delphi survey 2 κ statistic^	Delphi survey 3 κ statistic^
**All eleven mFI variables combined**		**0.728**	**0.811**	**0.780**
**Individual mFI variables**				
1	Functional status - not independent	0.969	0.846	0.783
2	History of hypertension requiring medication	0.851	0.925	0.963
3	History of chronic obstructive pulmonary disease or pneumonia	0.742	0.827	0.827
4	History of impaired sensorium	0.748	0.806	0.766
5	History of Diabetes Mellitus	0.915	1.000	1.000
6	History of myocardial infarction	0.794	1.000	1.000
7	History of congestive heart failure	0.830	*	*
8	History of cerebrovascular accident with neurologic deficit	0.744	0.600	*
9	History of transient ischemic attack without neurologic deficit	1.000	0.749	0.749
10	History of percutaneous coronary intervention, angina, or stenting	1.000	1.000	1.000
11	History of peripheral vascular disease or ischaemic rest pain	0.828	0.769	0.769

κ statistic [12]: 0–0.20 - no agreement 0.21–0.39 - minimal agreement 0.40–0.59 - weak agreement 0.60–0.79 - moderate agreement 0.80–0.90 - strong agreement

> 0.90 - almost perfect agreement

^ The p-value was < 0.001 for all

* No statistics was computed

## Discussion

### Key findings

We conducted a quantitative Delphi survey to define, by consensus, the ICD-10 variables required to estimate the modified frailty index (mFI). Overall, the Delphi survey reached a consensus for 87.5% of the ICD-10 variables from the 21-panel members who completed all three rounds. There was moderate to a strong inter-rater agreement between intensivists and geriatricians between the three surveys. From an original total of 128 codes, we were able to identify 54 ICD-10 codes, which intensivists and geriatricians considered necessary to estimate the mFI. These ICD-10 codes can be further investigated for their validity in estimating frailty in the intensive care or geriatric setting.

### Relationship to previous studies

The consensus methodology has been used to define the components of frailty assessment using the Delphi process in previous studies [14–16]. However, to our knowledge, this was the first study that attempted to obtain a consensus between experienced intensivists and geriatricians in identifying the most appropriate ICD-10 codes which could be used to estimate the mFI.

The issue of finding the appropriate ICD-10 codes needed to estimate the mFI is important, to potentially make an mFI equivalent globally available. Brazilian ICUs incorporated the mFI directly into their commercial ICU system (Epimed Monitor). This system is a database with a specific structured library of diagnoses and comorbidities. It has the capability of recording previous functional capacity based on Eastern Cooperative Oncology Group (ECOG) Performance Status and previously impaired sensorium [8, 17, 18]. This approach is not only impractical in real-time but also expensive, and therefore not generalizable on a global scale. Another recent post-hoc study, from a multicentre study [19], used a relatively small sample size from a single-centre [20], mapped the mFI variables from their large ICU Frailty database, and found that the mFI predicted hospital mortality [19]. However, these databases are not readily available and, therefore, not globally applicable, highlighting the need for an ICD-10 codes-based system and the rationale for our study.

The use of ICD-10 codes to screen frailty is a reasonably well-researched area. A recent systematic review [21] listed five models that have demonstrated validity, namely: electronic frailty index [22], hospital frailty risk score (HFRS) [6], frailty risk score [23], preoperative frailty index [24], and Dr. Foster global frailty score (FGFS) [25]. Two of these, HFRS and FGFS have been externally validated [21]. Although the ICD-10 codes used in our study may not cover all the concepts of the HFRS and FGFS, the mFI is a validated frailty screening tool. In our study, we selected all the pertinent ICD-10 codes to reflect the mFI items. The use of ICD-10 codes has several caveats. There may be a geographic and temporal variation in the coding [26]. Furthermore, these models should consider the amount of historic data that is required [27]. Consequently, it is possible that despite the Delphi consensus that was demonstrated in our study, there may be uncertainty if this can effectively identify frailty. Future studies should aim to validate the diagnostic and predictive ability to use ICD-10 codes to estimate an mFI equivalent.

### Implications

Our findings imply that intensivists and geriatricians could achieve consensus in determining which ICD-10 codes can be used to estimate the mFI. These 54 ICD-10 codes could act as surrogates and be used to estimate the mFI. As ICD-10 coding is globally available and free, its use could then provide global frailty data in large populations at a national or multinational level.

### Strengths and limitations

The study has several strengths. First, an expert consensus was used to select the items. Second, the response rate was 100% from all panel members in all three surveys. This response rate limits responder bias. Third, the Delphi panel included senior experienced clinicians. Fourth, the anonymity of the Delphi panel members was preserved throughout. Fifth, the inter-rater agreement was consistently between moderate and almost perfect agreement.

We acknowledge several limitations. The use of Delphi techniques of surveying experts may be considered inferior to evidence-based methods [28]. However, the ICD-10 quantification to estimate the 11 mFI variables is a novel and logical approach. Equally, consensus does not necessarily mean correctness and there is a lack of consensus on the optimum panel size or criteria for the termination [29]. These models built on these consensus findings will require robust evaluation and validation on clinical data sets [14] and compared with validated scores frailty screening tools such as the CFS [19, 30]. Also, even though clinical coders or medical statisticians are very familiar with ICD-10 coding, we only chose clinicians as panel members. Furthermore, the selection of panel ‘experts’ has been challenged as being subjective [31]. We did not provide an option for the Delphi panel members to suggest any relevant ICD-10 codes for the included comorbidities. This may be a source of bias; however, the ICD-10 codes are related to comorbidities for these eleven mFI variables and therefore appear reasonable. Finally, the DRG coding may sometimes be inaccurate with complications that occur during a hospital stay incorrectly coded as comorbidities [32, 33].

## Conclusion

This quantitative Delphi survey achieved consensus for which ICD-10 codes are appropriate as surrogates to estimate the mFI. There was moderate to a strong inter-rater agreement amongst intensivists and geriatricians participating in the study. Future studies should focus on validating the diagnostic and predictive value of using ICD-10 codes to estimate an mFI equivalent.

## Supplementary Information

**Additional file 1.**

**Additional file 2.**

## Data Availability

Not applicable. This was a Delphi survey. The 3 Delphi surveys are attached in the Supplementary appendix.
