# Supplementary material for: Defining ICD-10 surrogate variables to estimate the modified frailty index: a Delphi-based approach
Source: BMC Geriatr. 2022 May 13;22:422. doi: 10.1186/s12877-022-03063-x (PMC9107186; doi:10.1186/s12877-022-03063-x)
Supplement: Supplementary file 1 — Additional file 1. [file 12877_2022_3063_MOESM1_ESM.docx]

**Legends for Supplementary Tables**

**Supplementary Table 1:** The eleven modified frailty index (mFI) Variables.

**Supplementary Table 2:** The 128 ICD-10 codes included for the 11 mFI variables, that were included in Delphi Survey 1.

**Supplementary Table 3:** Delphi 1 survey, where respondents were requested to mark the items on a 3-point Likert scale. (‘yes’, ‘no’ and ‘maybe’). Numbers highlighted in red depict excluded variables after each Delphi round, while numbers in green depict included ICD-10 variables. Both red and green highlighted numbers were not carried to the next survey.

**Supplementary Table 4:** Delphi 2 survey: Respondents were requested to mark the items on a two-point Likert scale. (‘yes’ or ‘no’). Numbers highlighted in red depict excluded variables after each Delphi round, while numbers in green depict included ICD-10 variables. Both, the red and green highlighted numbers, were not carried forward to the next survey.

**Supplementary Table 5:** Delphi Survey 3, where respondents were requested to mark the items on a 5-point Likert scale: ‘strongly disagree’, ‘disagree’, ‘neutral’, ‘agree’ and, ‘strongly agree’. These responses were dichotomised to produce percentages: ‘strongly agree’, ‘agree’, and ‘neutral’ responses were grouped into one, and ‘disagree’ and ‘strongly disagree’ into another. Fisher’s exact test was used for group comparisons between intensivists and geriatricians. Numbers highlighted in red depict excluded variables after each Delphi round, while numbers in green depict included ICD-10 variables.

**Supplementary Table 6:** The 16 ICD-10 items that did not reach the 75% consensus between Geriatricians and Intensivists after the Delphi Survey 3, where respondents were requested to mark the items on a 5-point Likert scale: ‘strongly disagree’, ‘disagree’, ‘neutral’, ‘agree’ and ‘strongly agree’. These responses were dichotomised to produce percentages: ‘strongly agree’, ‘agree’, and ‘neutral’ responses were grouped into one, and ‘disagree’ and ‘strongly disagree’ into another. Fisher’s exact test was used for group comparisons between intensivists and geriatricians. Numbers highlighted in red depict if the Likert score was <75%, while numbers in green depict if the Likert score was >75%, ICD-10 variables.

**Supplementary Appendix:** mFI Delphi Surveys 1-3.
