# Supplementary material for: Defining ICD-10 surrogate variables to estimate the modified frailty index: a Delphi-based approach
Source: BMC Geriatr. 2022 May 13;22:422. doi: 10.1186/s12877-022-03063-x (PMC9107186; doi:10.1186/s12877-022-03063-x)
Supplement: Supplementary file 2 — Additional file 2. [file 12877_2022_3063_MOESM2_ESM.pdf]

mFI Delphi 1

Defining the ICD-10 variables required to calculate the modified frailty index using the Delphi Consensus: Survey-1

My name is Dr Ashwin Subramaniam. I am an intensive care specialist from Frankston Hospital, currently undertaking a PhD (Quantifying frailty in the critically ill) through Monash University.

Lead Investigator: Dr Ashwin Subramaniam
Associate Investigators: Ryo Ueno, Ravindranath Tiruvoipati, Jai Darvall, Velandai Srikanth, Michael Bailey, Rinaldo Bellomo, and David Pilcher

Purpose: To calculate the eleven co-morbidities modified frailty index (mFI) using the pertinent ICD-10 codes required to identify each of the eleven co-morbidities as an approximation. The ICD-10 codes were obtained from the Australian-Refined Diagnosis Related Groups (AR-DRG) upon patients' discharge from the hospital. We have included all possible ICD-10 codes pertinent to each of the 11 mFI variables.

Delphi Panel Members: On behalf of my co-authors, I request your help in this Delphi consensus process. You are one of the intensive care specialists and/or Geriatricians to be chosen as panel members. There may be about 3 to 4 surveys over the coming weeks, till we reach an 75% consensus for the possible ICD-10 codes that are absolutely required for each of the 11 mFI variables. I am seeking your help in this Delphi consensus process.

Delphi Survey: This is the first survey.

We need your help: To establish an appropriate set of ICD-10 codes that could be used in the mFI evaluation.

What it entails: For all of the survey questions, we have asked if the listed ICD-10 codes could be used to represent the mFI variable. We have also provided the actual definition that was used in NSQIP database. Kindly complete the survey by answering YES, NO or MAYBE against each ICD-10 variable.

Your participation: Participation is voluntary. By participating, you are providing consent for the study results to be used for publication. You will not receive any monetary benefits. You will be acknowledged in the manuscript as a Delphi Panel Member.

Only the researchers will be able to link your responses to your email address.

Time taken to complete the survey: Approximately 15-20 minutes

Background: Frailty, across adult age spectrum, is common in patients admitted to intensive care units (ICU), and patients with frailty generally have poorer outcomes. The validated clinical frailty scale (CFS) is the most commonly used scale in ICU patients globally. Frailty index-based assessment may be difficult to deliver by the critical care team on a routine basis. Shorter modified versions have been validated in other countries. The modified frailty index (mFI) was created by mapping eleven comorbidity variables contained within the National Surgical Quality Improvement Program (NSQIP) database contained within the original 70-item Canadian Study of Health and Ageing Frailty Index. The mFI was originally created for patients having surgery, but subsequently found to be predictive of increased mortality and length of stay in a large Brazilian cohort of critically ill patients. The mFI is calculated by allocating one point for each of the eleven co-morbidities (that encompasses previous medical events and functional capacity). The mFI categorises patients as non-frail if score=0-2, or frail if score is ≥3.

The 11 variables that constitute the mFI are -
1. Functional status (not independent)
2. History of hypertension requiring medication
3. History of chronic obstructive pulmonary disease or pneumonia
4. History of impaired sensorium
5. History of diabetes mellitus
6. History of myocardial infarction
7. History of congestive heart failure
8. History of stroke with neurologic deficit
9. History of percutaneous angiographic intervention, angina or stenting
10. History of Peripheral vascular disease or ischemic rest pain
11. History of Transient ischaemic attack or stroke

However, nine of the eleven mFI items refer to medical co-morbidities that do not conform to the frailty 2011 expert consensus statement: "frailty is a multidimensional construct consisting of six domains (physical performance, gait speed, mobility, nutritional status, mental health, and cognition)". Moreover, two of the eleven mFI variables ('not independent of functional status' and 'impaired sensorium') did not have readily available ICD-10 codes. We have included all possible ICD-10 codes pertinent to each of the 11 mFI variables. We aim to refine these codes to establish an appropriate set of ICD-10 codes that could be used in the mFI evaluation.

We have provided the actual NSQIP definitions that was used for every variable. However, if need further clarification, please feel free to click the link below for the detailed NSQIP data dictionary.

https://www.facs.org/~/media/Qles/quality%20programs/nsqip/nsqip_puf_userguide_2014.ashx

Please click the survey link here.

https://docs.google.com/forms/d/17C4ykWeVIm8Hy1JyNnXECMlmoX0vjv1JX1qYBdfHJ64/prefill

On behalf of my co-authors, I thank you immensely for helping out. If you have any queries, please contact me ashwin.subramaniam@monash.edu

Email *

Valid email address

This form is collecting email addresses. Change settings

I am an *

- Intensive Care Specialist
- Geriatrician

Years of experience in your current role *

- <5 years
- 5-10 years
- 10-15 years
- >15 years

Variable 1: Please advise if these ICD-10 codes represent "Functional status (not independent)". * ACTUAL definition of the variable as designed which are found in the NSQIP data dictionary. This variable focuses on the patient's abilities to perform activities of daily living (ADLs) in the 30 days prior to surgery. Activities of daily living are defined as 'the activities usually performed in the course of a normal day in a person's life'. ADLs include bathing, feeding, dressing, toileting, and mobility. The best functional status demonstrated by the patient in the preceding 30 days. 1. Partially dependent: The patient requires some assistance from another person for activities of daily living. This includes a person who utilizes prosthetics, equipment, or devices but still requires some assistance from another person for ADLs. 2. Totally dependent: The patient requires total assistance for all activities of daily living. Note: All patients with psychiatric illnesses should be evaluated for their ability to function with or without assistance with ADLs just as the non-psychiatric patient. For instance, if a patient with schizophrenia is able to care for him/herself without the assistance of nursing care, he/she is considered independent. If there is a change in the patient's functional status, (i.e., improvement to worsening) within the 30 days prior to surgery, report the patient's best functional status.

	YES	NO	Maybe
Z73 – life management ...	○	○	○
Z73.6 – ADL dysfunction	○	○	○
Z74 – Care-provider dep ...	○	○	○
Z74.1 – Need for assista ...	○	○	○
Z00-Z99 – problems/fac ...	○	○	○
R26.0-R26.9 – Gait probl ...	○	○	○
R27.0-R27.9 – Lack of c ...	○	○	○

R41 – Sarcopaenia	○	○	○
R41.81 – age-related co …	○	○	○
R54 – age-related physic …	○	○	○
W18 – Other fall on sam …	○	○	○
W19 – Unspecified fall	○	○	○
L89 – Decubitus ulcer	○	○	○
R32 – Urinary incontinen …	○	○	○
H54 – Blindness and low …	○	○	○
R33 – Urinary retention	○	○	○
S72 – fracture of femur	○	○	○

Variable 2: Please advise if these ICD-10 codes represent "History of hypertension requiring medication". ACTUAL definition of the variable as designed, which are found in the NSQIP data dictionary. Hypertension (HTN) 140/90 mmHg or above most of the time." The diagnosis of HTN must be documented in the patient's medical record and the condition is severe enough that it requires antihypertensive medication in the preceding 30 days. *

	YES	NO	Maybe
I10 – Essential hyperten...	○	○	○
I11 – Hypertensive heart...	○	○	○
I12 – Hypertensive kidne...	○	○	○
I13 – Hypertensive heart...	○	○	○
I15 – Secondary hyperte...	○	○	○
O10 – Pre-existing hyper...	○	○	○
O11 – Pre-eclampsia su...	○	○	○
O13 – Gestational hyper...	○	○	○
O14 – Pre-eclampsia	○	○	○
O15 – Eclampsia	○	○	○
I27 – Other pulmonary h...	○	○	○
I27.0 – primary pulmona...	○	○	○
I27.2 – Other secondary ...	○	○	○
U82.3 Hypertension	○	○	○

Variable 3: Please advise if these ICD-10 codes represent "History of chronic obstructive pulmonary disease or pneumonia".
ACTUAL definition of the variable as designed, which are found in the NSQIP data dictionary.
Medical record must document that there is a historical or current diagnosis of COPD AND at least one of the following, in the preceding 30 days: Functional disability from COPD (e.g., dyspnea, inability to perform ADLs) Or Requires chronic bronchodilator therapy with oral or inhaled agents or other medication specifically targeted to this disease

Multiple-choice grid

Rows

1. J44 – COPD
2. J09-J11 Influenza
3. J67 – Hypersensitivity pneumonitis
4. M05 – Rheumatoid lung disease
5. J12 – viral pneumonia
6. J13 – pneumonia due to streptococcal pn ...
7. J14 – pneumonia due to Haemophilus infl ...
8. J15 – Bacterial pneumonia, not elsewher ...
9. J16 – Pneumonia due to other infectious ...
10. J17 – Pneumonia in diseases classified ...
11. J18 – Pneumonia, organism unspecified
12. J43 – Emphysema
13. J47 - Bronchiectasis
14. J68 – Respiratory conditions due to inha ...
15. J69 – Pneumonitis due to solids and liq ...

Columns

- YES
- NO
- Maybe
- Add column

16. J70 – Respiratory conditions due to oth... ×
17. J84 – Other interstitial pulmonary disea... ×
18. J85 – Abscesses of lung and mediastin... ×
19. J90 – Pleural effusion ×
20. J95 – Intraoperative and postprocedural... ×
21. J96 – respiratory failure ×
22. P23 – Congenital pneumonia ×
23. U83 – Diseases of the respiratory system ×
24. R06 - Abnormalities in breathing ×
25. J98 – Other respiratory disorders ×
26. Add row

Variable 4: Please advise if these ICD-10 codes represent "History of impaired sensorium". ACTUAL definition of the variable as designed, which are found in the NSQIP data dictionary. "YES" is entered if patient is acutely confused and/or delirious and responds to verbal and/or mild tactile stimulation. Patient is noted to have developed an impaired sensorium if they have mental status changes, and/or delirium in the context of the current illness. Patients with chronic or long-standing mental status changes secondary to chronic mental illness (e.g., schizophrenia) or chronic dementing illnesses (e.g., multi-infarct dementia, senile dementia of the Alzheimer's type) are not included. *

	YES	NO	Maybe
F06 – Memory disturban...	○	○	○
G40.2 – Memory loss du...	○	○	○
G30 – Alzheimer’s Deme...	○	○	○
F01 – Vascular Dementia	○	○	○
A81.0 - CJD	○	○	○

G20 - Parkinson’s disease B22 - HIV

F04 – Organic Amnesia …

F10 – Amnesia related t…

F11-F19 – Amnesia relat… F05 –

Delirium

F00-F03 – Delirium supe…

F10.4 – Delirium tremens

F30-F39 – Mood Disorder…

F20-F29 – Schizophrenia

F40-F48 – Neurotic disorder…

F50-F59 – Behavioural s… F60-F69 –

Personality di…

G01-G02 – Meningitis

G05 – Encephalitis-myelitis…

H25 – SENILE CATARACT

H26 – Other cataract

H90.3 – Sensorineural lo…

H90.3 – Conductive hear…

H90.6-H90.8 – Mixed he…

H54.9 – Vision impairment…

H35 – Macular degeneration	○	○	○
Z51 – Other medical care	○	○	○

Variable 5: Please advise if these ICD-10 codes represent "History of diabetes mellitus". *
ACTUAL definition of the variable as designed, which are found in the NSQIP data dictionary. The treatment regimen of the patient's chronic, long-term management (> 2 weeks). Diabetes mellitus requiring daily dosages of exogenous parenteral insulin or a non-insulin anti-diabetic agent to prevent a hyperglycemia/metabolic acidosis. Patients whose diabetes are controlled by diet alone are not included.

	YES	NO	Maybe
E09 - Intermediate hyper ...	○	○	○
E10 - Type 1 diabetes m ...	○	○	○
E11 - Type 2 diabetes m ...	○	○	○
E13 – Other specified di ...	○	○	○
E14 - Unspecified diabetes	○	○	○
6O24. 410 - Gestational ...	○	○	○

Variable 6: Please advise if these ICD-10 codes represent "History of myocardial infarction". *
ACTUAL definition of the variable as designed, which are found in the NSQIP data dictionary.
"Yes" is entered for patients with a history of a non-Q wave or a Q wave infarct in the past six months as diagnosed in the patient's medical record.

	YES	NO	Maybe
I21 – Acute myocardial i ...	○	○	○
I22 – Subsequent myoca ...	○	○	○
I25 – Chronic ischaemic ...	○	○	○
Z86.7 – Personal history ...	○	○	○
Z82 - Family history of c ...	○	○	○
I51 – Complications and ...	○	○	○
I44 - Atrioventricular and ...	○	○	○
I49 - Other cardiac arrhyt ...	○	○	○
I45 - Other conduction di ...	○	○	○
R00 – Abnormalities in h ...	○	○	○

Variable 7: Please advise if these ICD-10 codes represent "History of congestive heart failure". * ACTUAL definition of the variable as designed, which are found in the NSQIP data dictionary. Only newly diagnosed CHF within the previous 30 days or a diagnosis of chronic CHF with new signs or symptoms in the 30 days prior to surgery fulfills this definition.

	YES	NO	Maybe
I50 – Heart failure	○	○	○
P29 – Cardiovascular di ...	○	○	○
U80.2 – Chronic heart fa ...	○	○	○
I34 - Non-rheumatic mitr ...	○	○	○
I35 - Non-rheumatic aort ...	○	○	○
R60 – Oedema	○	○	○

Variable 8: Please advise if these ICD-10 codes represent "History of stroke with neurologic deficit". ACTUAL definition of the variable as designed, which are found in the NSQIP data dictionary. "YES" is entered if patient has sustained acute or chronic neuromuscular injury resulting in total or partial paralysis or paresis (weakness) of one side of the body. 'YES' is entered if the patient has hemiplegia/hemiparesis (that has not recovered or been rehabilitated) upon arrival to the OR. "YES" is entered, if there is hemiplegia or hemiparesis associated with a CVA/Stroke also. *

	YES	NO	Maybe
I61 – Intracerebral haem ...	○	○	○
I63 – Cerebral infarction	○	○	○
I69 – Sequelae of cerebr ...	○	○	○
U80 – Diseases of the n ...	○	○	○
G00-G09 – Inflammatory ...	○	○	○
G20-G26 – Extrapyramid ...	○	○	○
G22 – Parkinsonism	○	○	○
G07 – Intracranial absce ...	○	○	○

Variable 9: Please advise if these ICD-10 codes represent "History of Transient ischaemic attack or stroke without neurological deficit". ACTUAL definition of the variable as designed, which are found in the NSQIP data dictionary. "YES" is entered if patient has transient ischemic attacks (TIAs). TIAs are focal neurologic deficits (e.g. numbness of an arm or amaurosis fugax) of sudden onset and brief duration (usually <30 minutes) that usually reflects dysfunction in a cerebral vascular distribution. These attacks may be recurrent and, at times, may precede a stroke. *

	YES	NO	Maybe
G45 – Transient cerebral ...	○	○	○
I64 – Stroke, not specifi ...	○	○	○

Variable 10: Please advise if these ICD-10 codes represent "History of percutaneous angiographic intervention, angina or stenting". ACTUAL definition of the variable as designed, which are found in the NSQIP data dictionary. "YES" is entered for patient who have undergone percutaneous coronary intervention (PCI) at any time (including any attempted PCI). This includes either balloon dilatation or stent placement. This does not include valvuloplasty procedures. *

	YES	NO	Maybe
I20 – Angina pectoris	◯	◯	◯
Z95 – Presence of cardi ...	◯	◯	◯
I71 - Aortic aneurysm an ...	◯	◯	◯

Variable 11: Please advise if these ICD-10 codes represent "History of Peripheral vascular disease or ischemic rest pain". ACTUAL definition of the variable as designed, which are found in the NSQIP data dictionary. "YES" is entered for a patient with any type of angioplasty (including stent placement) or revascularization procedure for atherosclerotic peripheral vascular disease (PVD) (e.g., aorta-femoral, femoral-femoral, femoral-popliteal) or a patient who has had any type of amputation procedure for PVD (e.g., toe amputations, transmetatarsal amputations, below the knee or above the knee amputations). Patients who have had amputation for trauma or a resection of abdominal aortic aneurysms should not be included. *

	YES	NO	Maybe
I70.2 - Atherosclerosis o ...	○	○	○
I73.9 - Arteriospasm/An ...	○	○	○
I77.9 - Peripheral arterial ...	○	○	○
I77.1 - Obliterative perip ...	○	○	○
I99.9 - Angiopathy	○	○	○
Z89 - amputation	○	○	○
I73 - Peripheral vascular ...	○	○	○
G46.8 - Other vascular s ...	○	○	○
T87 - complications fro ...	○	○	○

I am an *

○ Intensive Care Specialist

○ Geriatrician

Years of experience in your current role *

- <5 years
- 5-10 years
- 10-15 years
- >15 years

Variable 1: Please advise if these ICD-10 codes represent "Functional status (not independent)". * ACTUAL definition of the variable as designed which are found in the NSQIP data dictionary. This variable focuses on the patient's abilities to perform activities of daily living (ADLs) in the 30 days prior to surgery. Activities of daily living are defined as ‘the activities usually performed in the course of a normal day in a person’s life’. ADLs include bathing, feeding, dressing, toileting, and mobility. The best functional status demonstrated by the patient in the preceding 30 days. 1. Partially dependent: The patient requires some assistance from another person for activities of daily living. This includes a person who utilizes prosthetics, equipment, or devices but still requires some assistance from another person for ADLs. 2. Totally dependent: The patient requires total assistance for all activities of daily living. Note: All patients with psychiatric illnesses should be evaluated for their ability to function with or without assistance with ADLs just as the non-psychiatric patient. For instance, if a patient with schizophrenia is able to care for him/herself without the assistance of nursing care, he/she is considered independent. If there is a change in the patient’s functional status, (i.e., improvement to worsening) within the 30 days prior to surgery, report the patient’s best functional status.

	YES	NO	Maybe
Z73 – life management ...	○	○	○
Z73.6 – ADL dysfunction	○	○	○
Z74 – Care-provider dep ...	○	○	○
Z74.1 – Need for assista ...	○	○	○
Z00-Z99 – problems/fac ...	○	○	○
R26.0-R26.9 – Gait probl ...	○	○	○

R27.0-R27.9 – Lack of c …	◯	◯	◯
R41 – Sarcopaenia	◯	◯	◯
R41.81 – age-related co …	◯	◯	◯
R54 – age-related physic …	◯	◯	◯
W18 – Other fall on sam …	◯	◯	◯
W19 – Unspecified fall	◯	◯	◯
L89 – Decubitus ulcer	◯	◯	◯
R32 – Urinary incontinen …	◯	◯	◯
H54 – Blindness and low …	◯	◯	◯
R33 – Urinary retention	◯	◯	◯
S72 – fracture of femur	◯	◯	◯

Variable 2: Please advise if these ICD-10 codes represent "History of hypertension requiring medication". ACTUAL definition of the variable as designed, which are found in the NSQIP data dictionary. Hypertension (HTN) 140/90 mmHg or above most of the time." The diagnosis of HTN must be documented in the patient's medical record and the condition is severe enough that it requires antihypertensive medication in the preceding 30 days. *

	YES	NO	Maybe
I10 – Essential hyperten ...	○	○	○
I11 – Hypertensive heart ...	○	○	○
I12 – Hypertensive kidne ...	○	○	○
I13 – Hypertensive heart ...	○	○	○
I15 – Secondary hyperte ...	○	○	○
O10 – Pre-existing hyper ...	○	○	○
O11 – Pre-eclampsia su ...	○	○	○
O13 – Gestational hyper ...	○	○	○
O14 – Pre-eclampsia	○	○	○
O15 – Eclampsia	○	○	○
I27 – Other pulmonary h ...	○	○	○
I27.0 – primary pulmona ...	○	○	○
I27.2 – Other secondary ...	○	○	○
U82.3 Hypertension	○	○	○

Variable 3: Please advise if these ICD-10 codes represent "History of chronic obstructive pulmonary disease or pneumonia". ACTUAL definition of the variable as designed, which are found in the NSQIP data dictionary. Medical record must document that there is a historical or current diagnosis of COPD AND at least one of the following, in the preceding 30 days: Functional disability from COPD (e.g., dyspnea, inability to perform ADLs) Or Requires chronic bronchodilator therapy with oral or inhaled agents or other medication specifically targeted to this disease Or Hospitalization in the past for treatment of COPD Or An FEV1 of <75% of predicted on a prior pulmonary function test (PFT). Patients whose only pulmonary disease is asthma, an acute and chronic inflammatory disease of the airways resulting in bronchospasm are not included. Patients with diffuse interstitial fibrosis, sarcoidosis, or silicosis are not included. *

	YES	NO	Maybe
J44 – COPD			
J09-J11 Influenza	○	○	○
J67 – Hypersensitivity p...	○	○	○
M05 – Rheumatoid lung ...	○	○	○
J12 – viral pneumonia	○	○	○
J13 – pneumonia due to...	○	○	○
J14 – pneumonia due to...	○	○	○
J15 – Bacterial pneumo...	○	○	○
J16 – Pneumonia due to...	○	○	○
J17 – Pneumonia in dise...	○	○	○
J18 – Pneumonia, organ...	○	○	○
J43 – Emphysema	○	○	○
J47 - Bronchiectasis	○	○	○
J68 – Respiratory condit...	○	○	○
J69 – Pneumonitis due t...	○	○	○
	○	○	○

J70 – Respiratory condit...	○	○	○
J84 – Other interstitial p...	○	○	○
J85 – Abscesses of lung...	○	○	○
J90 – Pleural effusion	○	○	○
J95 – Intraoperative and...	○	○	○
J96 – respiratory failure	○	○	○
P23 – Congenital pneum...	○	○	○
U83 – Diseases of the re...	○	○	○
R06 - Abnormalities in br...	○	○	○

Variable 4: Please advise if these ICD-10 codes represent "History of impaired sensorium". ACTUAL definition of the variable as designed, which are found in the NSQIP data dictionary. "YES" is entered if patient is acutely confused and/or delirious and responds to verbal and/or mild tactile stimulation. Patient is noted to have developed an impaired sensorium if they have mental status changes, and/or delirium in the context of the current illness. Patients with chronic or long-standing mental status changes secondary to chronic mental illness (e.g., schizophrenia) or chronic dementing illnesses (e.g., multi-infarct dementia, senile dementia of the Alzheimer's type) are not included. *

	YES	NO	Maybe
F06 – Memory disturban...	○	○	○
G40.2 – Memory loss du...	○	○	○
G30 – Alzheimer's Deme...	○	○	○
F01 – Vascular Dementia	○	○	○
A81.0 - CJD	○	○	○
G20 - Parkinson's disease	○	○	○
B22 - HIV	○	○	○

F04 – Organic Amnesia ...

F10 – Amnesia related t...

F11-F19 – Amnesia relat... F05 –

Delirium

F00-F03 – Delirium supe...

F10.4 – Delirium tremens

F30-F39 – Mood Disorde...

F20-F29 – Schizophrenia

F40-F48 – Neurotic disor...

F50-F59 – Behavioural s...

F60-F69 – Personality di...

G01-G02 – Meningitis

G05 – Encephalitis-myeli... H25 –

SENILE CATARACT

H26 – Other cataract

H90.3 – Sensorineural lo...

H90.3 – Conductive hear...

H90.6-H90.8 – Mixed he...

H54.9 – Vision impairme...

H35 – Macular degenera... Z51 –

Other medical care

Variable 5: Please advise if these ICD-10 codes represent "History of diabetes mellitus". ACTUAL definition of the variable as designed, which are found in the NSQIP data dictionary. The treatment regimen of the patient's chronic, long-term management (> 2 weeks). Diabetes mellitus requiring daily dosages of exogenous parenteral insulin or a non-insulin anti-diabetic agent to prevent a hyperglycemia/metabolic acidosis. Patients whose diabetes are controlled by diet alone are not included. *

	YES	NO	Maybe
E09 - Intermediate hyper ...	○	○	○
E10 - Type 1 diabetes m ...	○	○	○
E11 - Type 2 diabetes m ...	○	○	○
E13 – Other specified di ...	○	○	○
E14 - Unspecified diabet ...	○	○	○
6O24. 410 - Gestational ...	○	○	○

Variable 6: Please advise if these ICD-10 codes represent "History of myocardial infarction". *
ACTUAL definition of the variable as designed, which are found in the NSQIP data dictionary. "Yes" is entered for patients with a history of a non-Q wave or a Q wave infarct in the past six months as diagnosed in the patient's medical record.

	YES	NO	Maybe
I21 – Acute myocardial i ...	○	○	○
I22 – Subsequent myoca ...	○	○	○
I25 – Chronic ischaemic ...	○	○	○
Z86.7 – Personal history ...	○	○	○
Z82 - Family history of c ...	○	○	○
I51 – Complications and ...	○	○	○
I44 - Atrioventricular and ...	○	○	○
I49 - Other cardiac arrhyt ...	○	○	○
I45 - Other conduction di ...	○	○	○
R00 – Abnormalities in h ...	○	○	○

Variable 7: Please advise if these ICD-10 codes represent "History of congestive heart failure". * ACTUAL definition of the variable as designed, which are found in the NSQIP data dictionary. Only newly diagnosed CHF within the previous 30 days or a diagnosis of chronic CHF with new signs or symptoms in the 30 days prior to surgery fulfills this definition.

	YES	NO	Maybe
I50 – Heart failure	○	○	○
P29 – Cardiovascular di ...	○	○	○
U80.2 – Chronic heart fa ...	○	○	○
I34 - Non-rheumatic mitr ...	○	○	○
I35 - Non-rheumatic aort ...	○	○	○
R60 – Oedema	○	○	○

Variable 8: Please advise if these ICD-10 codes represent "History of stroke with neurologic deficit". ACTUAL definition of the variable as designed, which are found in the NSQIP data dictionary. "YES" is entered if patient has sustained acute or chronic neuromuscular injury resulting in total or partial paralysis or paresis (weakness) of one side of the body. 'YES' is entered if the patient has hemiplegia/hemiparesis (that has not recovered or been rehabilitated) upon arrival to the OR. "YES" is entered, if there is hemiplegia or hemiparesis associated with a CVA/Stroke also. *

	YES	NO	Maybe
I61 – Intracerebral haem ...	◯	◯	◯
I63 – Cerebral infarction	◯	◯	◯
I69 – Sequelae of cerebr ...	◯	◯	◯
U80 – Diseases of the n ...	◯	◯	◯
G00-G09 – Inflammatory ...	◯	◯	◯
G20-G26 – Extrapyramid ...	◯	◯	◯
G22 – Parkinsonism	◯	◯	◯
G07 – Intracranial absce ...	◯	◯	◯

Variable 9: Please advise if these ICD-10 codes represent "History of Transient ischaemic attack or stroke without neurological deficit". ACTUAL definition of the variable as designed, which are found in the NSQIP data dictionary. "YES" is entered if patient has transient ischemic attacks (TIAs). TIAs are focal neurologic deficits (e.g. numbness of an arm or amaurosis fugax) of sudden onset and brief duration (usually <30 minutes) that usually reflects dysfunction in a cerebral vascular distribution. These attacks may be recurrent and, at times, may precede a stroke. *

	YES	NO	Maybe
G45 – Transient cerebral ...	◯	◯	◯
I64 – Stroke, not specifi ...	◯	◯	◯

Variable 10: Please advise if these ICD-10 codes represent "History of percutaneous angiographic intervention, angina or stenting". ACTUAL definition of the variable as designed, which are found in the NSQIP data dictionary. "YES" is entered for patient who have undergone percutaneous coronary intervention (PCI) at any time (including any attempted PCI). This includes either balloon dilatation or stent placement. This does not include valvuloplasty procedures. *

	YES	NO	Maybe
I20 – Angina pectoris	○	○	○
Z95 – Presence of cardi ...	○	○	○
I71 - Aortic aneurysm an ...	○	○	○

Variable 11: Please advise if these ICD-10 codes represent "History of Peripheral vascular disease or ischemic rest pain". ACTUAL definition of the variable as designed, which are found in the NSQIP data dictionary. "YES" is entered for a patient with any type of angioplasty (including stent placement) or revascularization procedure for atherosclerotic peripheral vascular disease (PVD) (e.g., aorta-femoral, femoral-femoral, femoral-popliteal) or a patient who has had any type of amputation procedure for PVD (e.g., toe amputations, transmetatarsal amputations, below the knee or above the knee amputations). Patients who have had amputation for trauma or a resection of abdominal aortic aneurysms should not be included. *

	YES	NO	Maybe
I70.2 - Atherosclerosis o ...	○	○	○
I73.9 - Arteriospasm/An ...	○	○	○
I77.9 - Peripheral arterial ...	○	○	○
I77.1 - Obliterative perip ...	○	○	○
I99.9 - Angiopathy	○	○	○
Z89 - amputation	○	○	○
I73 - Peripheral vascular ...	○	○	○
G46.8 - Other vascular s ...	○	○	○
T87 - complications fro ...	○	○	○

Defining the ICD-10 variables required to calculate the modified frailty index using the Delphi Consensus: Survey-2

My name is Dr Ashwin Subramaniam. I am an intensive care specialist from Frankston Hospital, currently undertaking a PhD (Quantifying the impact of frailty in the critically ill) through Monash University.

Lead Investigator: Dr. Ashwin Subramaniam
Associate Investigators: Ryo Ueno, Ravindranath Tiruvoipati, Jai Darvall, Velandai Srikanth, Michael Bailey, Rinaldo Bellomo, and David Pilcher

Purpose: To calculate the eleven co-morbidities modified frailty index (mFI) using the pertinent ICD-10 codes required to identify each of the eleven co-morbidities as an approximation. The ICD-10 codes were obtained from the Australian-Refined Diagnosis Related Groups (AR-DRG) upon patients' discharge from the hospital. We have included all possible ICD-10 codes pertinent to each of the 11 mFI variables.

Thank you for completing the Delphi Survey 1. We had a total of 21 responses from 13 Intensive care specialists and 8 Geriatricians. The collated results demonstrated only a 25% consensus. There were no statistical differences in the responses between geriatricians and intensivists. Please click this link to access the results of Delphi Survey 1.
https://docs.google.com/spreadsheets/d/1OAlAi941AkkLcLjQfkVaa5ybjofqQK0xyQGgH7YONBM/edit#gid=1777197197

I've excluded those ICD-10 codes for this survey.

Many have answered 'Maybe'. In Delphi Round 2 I have removed the 'Maybe' option. We are hoping for a 'YES' and 'NO' from you. Please mark these ICD-10 codes as variables that represent a comorbidity variable rather than as a frailty marker.

As Delphi Panel Members, on behalf of my co-authors, I request your help again.

Delphi Survey: This is the second survey.

Your participation is voluntary. By participating, you are providing consent for the study results to be used for publication. You will not receive any monetary benefits. You will be acknowledged in the manuscript as a Delphi Panel Member.

Only the researchers will be able to link your responses to your email address.

Time taken to complete the survey: Approximately 10-15 minutes

Background: Frailty, across the adult age spectrum, is common in patients admitted to intensive care units (ICU), and patients with frailty generally have poorer outcomes. The validated clinical frailty scale (CFS) is the most commonly used scale in ICU patients globally. Frailty index-based assessment may be difficult to deliver by the critical care team on a routine basis. Shorter modified versions have been validated in other countries. The modified frailty index (mFI) was created by mapping eleven comorbidity variables contained within the National Surgical Quality Improvement Program (NSQIP) database contained within the original 70-item Canadian Study of Health and Ageing Frailty Index. The mFI was originally created for patients having

surgery but subsequently found to be predictive of increased mortality and length of stay in a large Brazilian cohort of critically ill patients. The mFI is calculated by allocating one point for each of the eleven co-morbidities (that encompasses previous medical events and functional capacity). The mFI categorises patients as non-frail if score=0-2, or frail i the score is ≥3.

The 11 variables that constitute the mFI are -

1. Functional status (not independent)
2. History of hypertension requiring medication
3. History of chronic obstructive pulmonary disease or pneumonia
4. History of impaired sensorium
5. History of diabetes mellitus
6. History of myocardial infarction
7. History of congestive heart failure
8. History of stroke with neurologic deficit
9. History of percutaneous angiographic intervention, angina or stenting
10. History of Peripheral vascular disease or ischemic rest pain
11. History of Transient ischaemic attack or stroke

However, nine of the eleven mFI items refer to medical co-morbidities that do not conform to the frailty 2011 expert consensus statement: "frailty is a multidimensional construct consisting of six domains (physical performance, gait speed, mobility, nutritional status, mental health, and cognition)". Moreover, two of the eleven mFI variables (‘not independent of functional status’ and ‘impaired sensorium’) did no have readily available ICD-10 codes. We have included all possible ICD-10 codes pertinent to each of the 11 mFI variables. We aim to refine these codes to establish an appropriate set of ICD-10 codes that could be used in the mFI evaluation.

We have provided the actual NSQIP definitions that were used for every variable. However, if need further clarification, please feel free to click the link below for the detailed NSQIP data dictionary.

https://www.facs.org/~/media/Qles/quality%20programs/nsqip/nsqip_puf_userguide_2014.ashx

Please click the survey link here.

https://docs.google.com/forms/d/1yajIasT_MXtds3eQDijE8PebNGA8vxJU2yKeMhEX3lA/edit

On behalf of my co-authors, I thank you immensely for helping out. If you have any queries, please contact me ashwin.subramaniam@monash.edu *Required

1. Email *

2. Variable 1: Please advise if these ICD-10 codes represent "Functional status (not independent)". 2 ICD-10 codes that obtained 75% consensus has been removed. *

Mark only one oval per row.

	Yes	No
[Z73 – life management difficulty]	⬭	⬭
[Z74.1 – Need for assistance]	⬭	⬭
[Z00-Z99 – problems/factors influencing health status and contact with health services]	⬭	⬭
[R26.0- R26.9 – Gait problems]	⬭	⬭
[R27.0 -R27.9 – Lack of coordination]	⬭	⬭
[R41 – Sarcopaenia]	⬭	⬭
[R41.81 – age-related cognitive decline]	⬭	⬭
[R54 – age-related physical disability]	⬭	⬭
[W18 – Other fall on same level]	⬭	⬭
[W19 – Unspecified fall]	⬭	⬭
[L89 – Decubitus ulcer]	⬭	⬭
[R32 – Urinary incontinence]	⬭	⬭
[H54 – Blindness and low vision]	⬭	⬭
[R33 – Urinary retention]	⬭	⬭
[S72 – fracture of femur]	⬭	⬭

3. Variable 2: Please advise if these ICD-10 codes represent "History of hypertension requiring medication". 6 ICD-10 codes that obtained 75% consensus has been removed. *

Mark only one oval per row.

	YES	NO
I10 – Essential hypertension		
I15 – Secondary hypertension		
O10 – Pre-existing hypertension in pregnancy		
O11 – Pre-eclampsia superimposed on chronic hypertension		
O13 – Gestational hypertension		
I27.0 – primary pulmonary hypertension		
I27.2 – Other secondary pulmonary Hypertension		
U82.3 Hypertension		

4. Variable 3: Please advise if these ICD-10 codes represent "History of chronic obstructive pulmonary disease or pneumonia". 7 ICD-10 codes that obtained 75% consensus has been removed. *

Mark only one oval per row.

	YES	NO
J12 – viral pneumonia		
J13 – pneumonia due to streptococcal pneumoniae		
J14 – pneumonia due to Haemophilus influenzae]		
J15 – Bacterial pneumonia, not elsewhere classified		

J16 – Pneumonia due to other infectious organisms	⬭	⬭
J17 – Pneumonia in diseases classified elsewhere	⬭	⬭
J18 – Pneumonia, organism unspecified	⬭	⬭
J47 - Bronchiectasis	⬭	⬭
J68 – Respiratory conditions due to inhalation of chemicals, gases, fumes, and vapours	⬭	⬭
J69 – Pneumonitis due to solids and liquids	⬭	⬭
J70 – Respiratory conditions due to other external agents	⬭	⬭
J95 – Intraoperative and postprocedural disorders of respiratory system, not elsewhere	⬭	⬭
J96 – respiratory failure	⬭	⬭
P23 – Congenital pneumonia	⬭	⬭
U83 – Diseases of the respiratory system	⬭	⬭
R06 - Abnormalities in breathing	⬭	⬭
J98 – Other respiratory disorders	⬭	⬭

5. Variable 4: Please advise if these ICD-10 codes represent "History of impaired sensorium". ICD-10 codes that obtained 75% consensus has been removed. *

Mark only one oval per row.

	YES	NO
F06 – Memory disturbance	⬭	⬭
G40.2 – Memory loss due to seizures	⬭	⬭
G30 – Alzheimer's Dementia	⬭	⬭

F01 – Vascular Dementia		
A81.0 - CJD		
G20 - Parkinson’s disease		
F04 – Organic Amnesia Syndrome		
F10 – Amnesia related to Alcohol		
F11-F19 – Amnesia related to psychoactive agents		
F10.4 – Delirium tremens		
F50-F59 – Behavioural syndromes		
G01-G02 – Meningitis		
G05 – Encephalitis-myelitis		
H90.3 – Sensorineural loss		
H90.3 – Conductive hearing loss		
H90.6-H90.8 – Mixed hearing loss		
H54.9 – Vision impairment		
H35 – Macular degeneration		
Z51 – Other medical care		

6. Variable 5: Please advise if these ICD-10 codes represent "History of diabetes mellitus". 1 ICD-10 code that obtained 75% consensus has been removed. *

Mark only one oval per row.

	YES	NO
E09 - Intermediate hyperglycaemia	⬭	⬭
E11 - Type 2 diabetes mellitus	⬭	⬭
E13 – Other specified diabetes mellitus	⬭	⬭
E14 - Unspecified diabetes mellitus	⬭	⬭
6O24. 410 - Gestational diabetes mellitus in pregnancy, diet controlled	⬭	⬭

7. Variable 6: Please advise if these ICD-10 codes represent "History of myocardial infarction" ICD-10 codes that obtained 75% consensus has been removed. *

Mark only one oval per row.

	YES	NO
I25 – Chronic ischaemic heart disease	⬭	⬭
Z86.7 – Personal history of diseases of circulatory system	⬭	⬭
I51 – Complications and ill-defined descriptions of heart disease	⬭	⬭
I44 - Atrioventricular and left bundle branch block	⬭	⬭
I49 - Other cardiac arrhythmias	⬭	⬭
I45 - Other conduction disorders	⬭	⬭
R00 – Abnormalities in heartbeat	⬭	⬭

8. Variable 7: Please advise if these ICD-10 codes represent "History of congestive heart failure". 75% Consensus was not obtained in any of these ICD-10 codes. *

Mark only one oval per row.

	YES	NO
I50 – Heart failure	⬭	⬭
P29 – Cardiovascular disorders originating in the perinatal period	⬭	⬭
U80.2 – Chronic heart failure	⬭	⬭
I34 - Non-rheumatic mitral valve disorder	⬭	⬭
I35 - Non-rheumatic aortic valve disorder	⬭	⬭
R60 – Oedema	⬭	⬭

9. Variable 8: Please advise if these ICD-10 codes represent "History of stroke with a neurological deficit". 75% Consensus was not obtained in any of these ICD-10 codes. *

Mark only one oval per row.

	YES	NO
I61 – Intracerebral haemorrhage	⬭	⬭
I63 – Cerebral infarction	⬭	⬭
I69 – Sequelae of cerebrovascular disease	⬭	⬭
U80 – Diseases of the nervous system	⬭	⬭
G00-G09 – Inflammatory diseases of CNS	⬭	⬭
G20-G26 – Extrapyramidal syndrome	⬭	⬭
G22 – Parkinsonism	⬭	⬭
G07 – Intracranial abscess	⬭	⬭

10. Variable 9: Please advise if these ICD-10 codes represent "History of Transient ischaemic attack or stroke without neurological deficit". 1 ICD-10 code that obtained 75% consensus has been removed. *

Mark only one oval per row.

	YES	NO
I64 – Stroke, not specified as haemorrhage or infarction	⬭	⬭

11. Variable 10: Please advise if these ICD-10 codes represent "History of percutaneous angiographic intervention, angina or stenting". 75% Consensus was obtained in 1 ICD-10 code.
Mark only one oval per row.

	YES	NO
I20 – Angina pectoris	⬭	⬭
Z95 – Presence of cardiac and vascular implants and grafts	⬭	⬭

12. Variable 11: Please advise if these ICD-10 codes represent "History of Peripheral vascular disease or ischemic rest pain". 75% Consensus was not obtained in any of these ICD-10 codes. *
Mark only one oval per row.

	YES	NO
I70.2 - Atherosclerosis of arteries of extremities	⬭	⬭
I73.9 - Arteriospasm / Angiospasm	⬭	⬭
I77.9 - Peripheral arterial insufficiency	⬭	⬭
I77.1 - Obliterative peripheral arteries	⬭	⬭
I99.9 - Angiopathy	⬭	⬭
Z89 - amputation	⬭	⬭
I73 - Peripheral vascular disease	⬭	⬭
G46.8 - Other vascular syndromes of brain in cerebrovascular diseases	⬭	⬭
T87 - complications from amputation	⬭	⬭

mFI Delphi 3 Survey

Thank you so much for being a Delphi panel member and completing the Delphi 2 survey. All 21 responses from 13 Intensive care specialists and 8 Geriatricians were analysed. The collated results demonstrated almost 70% consensus. Please click this link to access the results of Delphi Survey 2.

As Delphi Panel Members, on behalf of my co-authors, I request your help again. I promise this will be the last survey.

Purpose: To calculate the eleven co-morbidities modified frailty index (mFI) using the pertinent ICD-10 codes required to identify each of the eleven co-morbidities as an approximation. The ICD-10 codes were obtained from the Australian-Refined Diagnosis Related Groups (AR-DRG) upon patients' discharge from the hospital. We have included all possible ICD-10 codes pertinent to each of the 11 mFI variables.

Delphi Survey: This is the third and final survey. We will accept a majority rule, for those variables that have not reached a consensus. I've only included the 38 variables that we do not have a consensus on yet.

Lead Investigator: Dr. Ashwin Subramaniam

Associate Investigators: Ryo Ueno, Ravindranath Tiruvoipati, Jai Darvall, Velandai Srikanth, Michael Bailey, David Pilcher, and Rinaldo Bellomo

In Delphi Round 3, I have introduced a 5-point Likert scale. Please mark these ICD-10 codes as variables that represent a comorbidity variable rather than as a frailty marker.

Your participation is voluntary. By participating, you are providing consent for the study results to be used for publication. You will not receive any monetary benefits. You will be acknowledged in the manuscript as a Delphi Panel Member.

Only the researchers will be able to link your responses to your email address.

The time taken to complete the survey is approximately 5-6 minutes

Background: Frailty, across the adult age spectrum, is common in patients admitted to intensive care units (ICU), and patients with frailty generally have poorer outcomes. The validated clinical frailty scale (CFS) is the most commonly used scale in ICU patients globally. Frailty index-based assessment may be difficult to deliver by the critical care team on a routine basis. Shorter modified versions have been validated in other countries. The modified frailty index (mFI) was created by mapping eleven comorbidity variables contained within the National Surgical Quality Improvement Program (NSQIP) database contained within the original 70-item Canadian Study of Health and Ageing Frailty Index. The mFI was originally created for patients having surgery but subsequently found to be predictive of increased mortality and length of stay in a large Brazilian cohort of critically ill patients. The mFI is calculated by allocating one point for each of the eleven co-morbidities (that encompasses previous medical events and

functional capacity). The mFI categorizes patients as non-frail if score=0-2, or frail i the score is ≥3.
The 11 variables that constitute the mFI are -

1. Functional status (not independent)
2. History of hypertension requiring medication
3. History of chronic obstructive pulmonary disease or pneumonia
4. History of impaired sensorium
5. History of diabetes mellitus
6. History of myocardial infarction
7. History of congestive heart failure
8. History of stroke with neurologic deficit
9. History of percutaneous angiographic intervention, angina or stenting
10. History of Peripheral vascular disease or ischemic rest pain
11. History of Transient ischaemic attack or stroke

The results of Delphi 2 survey is available in this link.
https://docs.google.com/spreadsheets/d/13esOImyoJydglp9RXv3mfrordEteaY6fInbRpDdWsUA/edit#g d=0

We have provided the actual NSQIP definitions that were used for every variable. However, if need further clarification, please feel free to click the link below for the detailed NSQIP data dictionary.

https://www.facs.org/~/media/Qles/quality%20programs/nsqip/nsqip_puf_userguide_2014.ashx

Please click the survey link here.

https://docs.google.com/forms/d/e/1FAIpQLSfnZitlvBPz7vEg7Q5JiNg7eb8AhyLmtNY5l2QBIiu_VRChw/viewform

On behalf of my co-authors, I thank you immensely for helping out. If you have any queries, please contact me ashwin.subramaniam@monash.edu *Required

1. Email *

2. Variable 1 - "Functional status (not independent)": Do you think this ICD-10 code should be used as a comorbidity marker to define the mFI variable? *

Mark only one oval per row.

	Strongly Agree	Agree	Neutral	Disagree	Strongly Disagree
Z73 – life management difficulty					
Z74.1 – Need for assistance					
Z00-Z99 – problems/factors influencing health status and contact with health services					
R26.0- R26.9 – Gait problems					
R27.0 -R27.9 – Lack of coordination					
R41 – Sarcopaenia					
R41.81 – age-related cognitive decline					
R54 – age-related physical disability					
W18 – Other fall on same level					
W19 – Unspecified fall					
L89 – Decubitus ulcer					
R32 – Urinary incontinence					
H54 – Blindness and low vision					
R33 – Urinary retention					

3. Variable 2 - "History of hypertension requiring medication": Do you think this ICD-10 code should be in used as a comorbidity marker to define the mFI variable? *

Mark only one oval per row.

	Strongly Agree	Agree	Neutral	Disagree	Strongly Disagree
O10 – Pre-existing hypertension in pregnancy					
O11 – Pre-eclampsia superimposed on chronic hypertension					
U82.3 Hypertension					

4. Variable 3 - "History of chronic obstructive pulmonary disease or pneumonia": Do you think this ICD-10 code should be in used as a comorbidity marker to define the mFI variable? *

Mark only one oval per row.

	Strongly Agree	Agree	Neutral	Disagree	Strongly Disagree
J47 - Bronchiectasis					
P23 – Congenital pneumonia					

5. Variable 4 - "History of impaired sensorium": Do you think this ICD-10 code should be in use as a comorbidity marker to define the mFI variable? *

Mark only one oval per row.

	Strongly Agree	Agree	Neutral	Disagree	Strongly Disagree
G40.2 – Memory loss due to seizures	⬭	⬭	⬭	⬭	⬭
G20 - Parkinson’s disease	⬭	⬭	⬭	⬭	⬭
F10 – Amnesia related to Alcohol	⬭	⬭	⬭	⬭	⬭
F11-F19 – Amnesia related to psychoactive agents	⬭	⬭	⬭	⬭	⬭
F10.4 – Delirium tremens	⬭	⬭	⬭	⬭	⬭
G05 – Encephalitis-myelitis	⬭	⬭	⬭	⬭	⬭
H90.3 – Sensorineural loss	⬭	⬭	⬭	⬭	⬭
H90.3 – Conductive hearing loss	⬭	⬭	⬭	⬭	⬭
H90.6-H90.8 – Mixed hearing loss	⬭	⬭	⬭	⬭	⬭
H54.9 – Vision impairment	⬭	⬭	⬭	⬭	⬭
H35 – Macular degeneration	⬭	⬭	⬭	⬭	⬭

6. Variable 5 - "History of diabetes mellitus": Do you think this ICD-10 code should be in used a a comorbidity marker to define the mFI variable? *

Mark only one oval per row.

	Strongly Agree	Agree	Neutral	Disagree	Strongly Disagree
6O24. 410 - Gestational diabetes mellitus in pregnancy, diet controlled	⬭	⬭	⬭	⬭	⬭

7. Variable 8 - "History of stroke with the neurological deficit": Do you think this ICD-10 code should be in used as a comorbidity marker to define the mFI variable? *

Mark only one oval per row.

	Strongly Agree	Agree	Neutral	Disagree	Strongly Disagree
I69 – Sequelae of cerebrovascular disease	⬭	⬭	⬭	⬭	⬭

8. Variable 9 - "History of TIA or stroke without neuro deficit": Do you think this ICD-10 code should be in used as a comorbidity marker to define the mFI variable? *
Mark only one oval per row.

	Strongly Agree	Agree	Neutral	Disagree	Strongly Disagree
I64 – Stroke, not specified as haemorrhage or infarction	⬭	⬭	⬭	⬭	⬭

9. Variable 10 - "History of PCI, angina or stenting": Do you think this ICD-10 code should be in used as a comorbidity marker to define the mFI variable? *
Mark only one oval per row.

	Strongly Agree	Agree	Neutral	Disagree	Strongly Disagree
I20 – Angina pectoris	⬭	⬭	⬭	⬭	⬭
Z95 – Presence of cardiac and vascular implants and grafts	⬭	⬭	⬭	⬭	⬭

10. Variable 11 - "History of Peripheral vascular disease or ischemic rest pain": Do you think thi ICD-10 code should be in used as a comorbidity marker to define the mFI variable? *

Mark only one oval per row.

	Strongly Agree	Agree	Neutral	Disagree	Strongly Disagree
I73.9 - Arteriospasm / Angiospasm	⬭	⬭	⬭	⬭	⬭
I99.9 - Angiopathy	⬭	⬭	⬭	⬭	⬭
Z89 - amputation	⬭	⬭	⬭	⬭	⬭

This content is neither created nor endorsed by Google.

Google Forms
